# Specific disruption of the ventral anterior temporo-frontal network reveals key implications for language comprehension and cognition

**DOI:** 10.1038/s42003-022-03983-9

**Published:** 2022-10-10

**Authors:** Evie Kourtidou, Dimitrios Kasselimis, Georgia Angelopoulou, Efstratios Karavasilis, Georgios Velonakis, Nikolaos Kelekis, Ioannis Zalonis, Ioannis Evdokimidis, Constantin Potagas, Michael Petrides

**Affiliations:** 1 National and Kapodistrian University of Athens, Aeginition Hospital, Neuropsychology and Language Disorders Unit, First Department of Neurology, 72-74 Vassilissis Sofias Ave, 11528 Athens, Greece; 2 Panteion University of Social and Political Sciences, Department of Psychology, 136 Andrea Syngrou Av., 17671 Athens, Greece; 3 University of Peloponnese, Department of Speech and Language Therapy, Antikalamos, 24100 Kalamata, Greece; 4 Democritus University of Thrace, Medical School, 6th kilometer, P.O. Box. 68100, Dragana, Alexandroupolis Greece; 5 National and Kapodistrian University of Athens, Aeginition Hospital “Attikon”, Second Department of Radiology, 1 Rimini St., 12462 Haidari, Greece; 6 National and Kapodistrian University of Athens, Aeginition Hospital, Neuropsychological Laboratory, First Department of Neurology, 72-74 Vassilissis Sofias Ave, 11528 Athens, Greece; 7 McGill University, Montreal Neurological Institute and Hospital, Department of Neurology and Neurosurgery, 3801 University Street, Montreal, Québec, QC H3A 2B4 Canada

**Keywords:** Language, Stroke, Stroke

## Abstract

Recent investigations have raised the question of the role of the anterior lateral temporal cortex in language processing (ventral language network). Here we present the language and overall cognitive performance of a rare male patient with chronic middle cerebral artery cerebrovascular accident with a well-documented lesion restricted to the anterior temporal cortex and its connections via the extreme capsule with the pars triangularis of the inferior frontal gyrus (i.e. Broca’s region). The performance of this unique patient is compared with that of two chronic middle cerebral artery cerebrovascular accident male patients with damage to the classic dorsal posterior temporo-parietal language system. Diffusion tensor imaging is used to reconstruct the relevant white matter tracts of the three patients, which are also compared with those of 10 healthy individuals. The patient with the anterior temporo-frontal lesion presents with flawless and fluent speech, but selective impairment in accessing lexico-semantic information, in sharp contrast to the impairments in speech, sentence comprehension and repetition observed after lesions to the classic dorsal language system. The present results underline the contribution of the ventral language stream in lexico-semantic processing and higher cognitive functions, such as active selective controlled retrieval.

## Abstract (web summary)

Neuropsychological profiling and clinical DTI of three stroke patients highlight the importance of the ventral language system in normal language comprehension and cognition.

## Introduction

Classic models of the neural basis of language postulate that, in the left language-dominant hemisphere, language function is mediated by a dorsal posterior temporal region (Wernicke’s area), damage of which leads to comprehension impairment, and its connections with a region in the posterior part of the inferior frontal gyrus (IFG) known as Broca’s area, where a lesion leads to impaired speech production with preserved comprehension^1^. In the classic standard model, the dorsal posterior temporal region is associated with language comprehension (Wernicke’s area), but the anterior temporal cortical region is not considered in relation to language processing^1^. In recent years, however, a number of investigations have proposed that the lateral part of the anterior temporal lobe (ATL), i.e. the cortex anterior to the classic posterior temporal region (Wernicke’s area), should also be considered a part of the language system in the language dominant left hemisphere^2–4^. The exact contribution of this left anterior temporal region to language processing has been debated. According to Patterson and colleagues^2^, the left ATL along with its counterpart in the right hemisphere, constitute a semantic hub that binds the semantic properties of words. Although this has been evident in patients with neurodegenerative diseases, such as primary progressive aphasia^4–6^ and semantic dementia^7^, the results of studies focusing on unilateral left ATL lesions appear contradictory. Lambon-Ralph et al.^8^ did not confirm deficits in semantic tasks in patients with unilateral left ATL lesions, and a unilateral left ATL lesion was also not related with significant deficits in mapping sound-to-meaning in patients with cerebrovascular lesions during the acute phase^9^. A lexical retrieval deficit was the only symptom observed in a patient who underwent left ATL resection^10^. Moreover, a specific and necessary role of the left ATL, and particularly the middle temporal gyrus (MTG), in mapping concepts to words was demonstrated in a large group of post-stroke aphasics by Schwartz and colleagues^3^. In the latter study, anterior MTG lesions were related to semantic error production. Similarly, Mesulam et al.^4^ reported a significant relationship between severe word comprehension impairment and left ATL atrophy in primary progressive aphasia patients.

Anatomical studies have shown that the anterior parts of the superior and middle temporal gyri (i.e. the parts anterior to the sulcus acousticus), which correspond to the anterior parts of Brodmann areas 22 and 21, are connected with the frontal cortex via a distinct white matter tract coursing through the extreme capsule, i.e. the temporo-frontal extreme capsule fasciculus (TFexcF)^11–15^. This connection targets various prefrontal areas, and in the IFG (Broca’s region) it targets primarily the pars triangularis (area 45)^11–15^. The TFexcF, which connects monosynaptically and bi-directionally anterior lateral temporal cortex with lateral frontal areas, including Broca’s region on the IFG, should not be confused with the uncinate fasciculus that connects the temporal polar and adjacent medial ATL (e.g., amygdala, pyriform and entorhinal cortex) with orbitofrontal and ventromedial frontal cortical areas^11–15^. The TFexcF should not be confused with the so-called inferior fronto-occipital fasciculus (IFOF) which is a multi-component and multi-step stream of fibers^16,17^. IFOF refers to a stream of fibers that connect occipital cortex with posterior inferior temporal cortex via various local connections and then the anterior temporal cortex to frontal cortex. The direct monosynaptic pathway, the TFexcF, that then links temporal cortex with lateral frontal cortex is often, incorrectly, included in reconstructions of the multi-component and multi-step fiber stream referred to as the IFOF. It is precisely for this reason that we refer to the monosynaptic fasciculus that connects lateral anterior temporal cortex to frontal cortex as the TFexcF to avoid the current confusion in the literature with the ill-defined multi-component IFOF. This confusion in terminology is important to note because, of course, it is possible to attribute incorrectly temporo-frontal interactions related to semantic processing with visual occipital interactions via the inferior fronto-occipital fasciculus. Thus, many of the so-called contributions of the IFOF to language processing are the result of incorrect definition of white matter pathways mixing the direct monosynaptic temporo-frontal fibers that we define as the TFexcF^11–15^ with the multi-component and multi-step fiber stream, the IFOF.

Thus, the anterior lateral temporal cortical region has its own distinct direct monosynaptic pathway (i.e. the TFexcF) connecting it with a distinct part of Broca’s region in the IFG, i.e. the pars triangularis where granular area 45 lies^11–15^, constituting the key component of the ventral network for language. On the other hand, the adjacent pars opercularis (area 44), is the area primarily connected with areas of the dorsal posterior language system located in the inferior parietal lobule and posterior temporal lobe. This dorsal network along with the aforementioned ventral network, comprise the dual stream for language^18–20^.

The classic dorsal posterior language system is considered to be involved primarily in the phonological sublexical processing of linguistic information, whereas the ventral temporal system has been related to semantic processing of linguistic information^19,20^. Evidence from both patients with primary progressive aphasia and cerebrovascular accidents (CVA) supports this dichotomy, relating lesion or atrophy in the dorsal system with deficits in the repetition of sentences and in the ventral system with lexico-semantic deficits^21,22^.

Here we should recall that electrical stimulation of the pars opercularis (area 44) of the IFG in alert patients undergoing brain surgery leads to pure speech arrest^23^. On the other hand, electrical stimulation of the pars triangularis (area 45) that lies immediately anterior to the opercular region does not produce the speech arrest that one observes after stimulation of the pars opercularis (area 44), but rather unspecified mild disturbances, such as word finding difficulties or hesitations in naming^23^. Clearly, the dysgranular area 44 on the pars opercularis is directly involved with planning the motor aspects of speech output (i.e. electrical stimulation results in speech arrest), but the role of the granular area 45 that lies just anterior on the pars triangularis of the IFG remains to be specified. Functional neuroimaging studies have linked the cortex on the pars triangularis (area 45) with higher cognitive processing and, in particular, the top-down controlled selective retrieval of verbal information^24^. However, to our knowledge, no previous study has examined a case of a lesion affecting selectively area 45, leaving the adjacent area 44 intact.

In the present investigation, we had the rare opportunity to examine in detail a CVA patient with damage restricted to the ventral anterior temporal network for language (AA), in comparison with two CVA patients with lesion restricted within the classical dorsal language system (MM and TA). Our study aimed to provide evidence regarding the distinct role of the ventral language system based on differential language and other cognitive performance, something that can only take place when rare cases with restricted lesions are examined in detail^25,26^.

Anatomical examination showed that AA’s lesion involved damage to the anterior parts of the superior and middle temporal gyri (without any damage to the dorsal posterior temporal gyrus and adjacent inferior parietal region) and, in the frontal lobe, there was only damage to the pars triangularis of the IFG (where granular area 45 lies), including the white matter connections between these areas via the TFexcF. However, area 44 (pars opercularis) was completely unaffected, providing a unique opportunity to explore the relative functional role in speech production of the pars triangularis in the left hemisphere.

The results shed light on the role of this key component of the ventral language system, i.e. pars triangularis of the IFG and its connections with areas of the ATL via TFexcF, underlining its involvement in lexico-semantic processing through a higher cognitive function, i.e. the active selective controlled retrieval of lexico-semantic information. Moreover, the role of area 45 in language and cognition is highlighted through a rare lesion that affects it while sparing its neighboring area 44.

## Results and discussion

### Case 1 (AA)

**Fig. 1:**
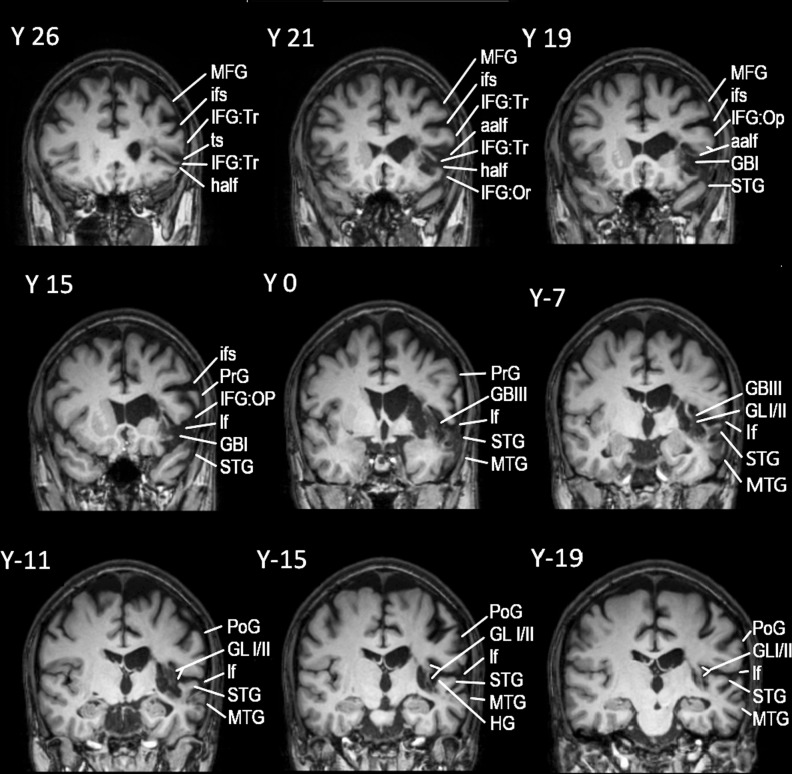
Successive coronal MRI images in MNI stereotaxic coordinates (Y) depicting the lesion of patient AA. The lesion appears at Y35 and continues as far as Y20, including a part of the pars triangularis (area 45) and the white matter below (see Y26, Y21). At Y19, the lesion also includes part of the caudate, the adjacent internal capsule (IC), the external capsule (EC), and the temporo-frontal extreme capsule fasciculus (TFexcF), just below insular gyrus Brevis I (GBI). Subcortically, there is damage to the claustrum, putamen, and the anterior part of the insula (GBI) under which courses the TFexcF. From Y15 to Y7, the lesion includes a small part of the anterior STG. Moving on posteriorly, between Y3 and Y-3, one can observe damage to the TFexcF which courses between the claustrum and the anterior insula (gyrus Brevis III; GBIII), the STG and a small part of MTG, including both banks of the superior temporal sulcus (sts) (see Y0). At Y-7, one observes that the lesion is located anterior to the level of the sulcus acousticus (sa) that lies on the lateral STG (see also Fig. 2). Subcortically, the lesion includes part of the caudate, IC, EC, TFexcF between the claustrum and insular gyrus Brevis III and gyrus Longus I & II, STG, sts and the whole MTG. The temporal lesion is visible as far posterior as Y-15 where it is restricted to the insula, caudate, putamen, claustrum, EC, TFexcF, and at Y-19 to putamen and lower insula, leaving Heschl’s gyrus and the surrounding temporal areas completely spared. Posterior to Y-20, no lesion is detected. Brain areas are topologically defined according to the atlas of the morphology of the human cerebral cortex in the MNI Stereotaxic Space^67^. aalf ascending anterior ramus of the lateral fissure; GBI gyrus brevis I of insula; GBIII gyrus brevis III of insula; GLI gyrus longus I of insula; GLII gyrus longus II of insula; half horizontal ascending ramus of the lateral fissure; HG Heschl’s gyrus; IFG:Tr inferior frontal gyrus, pars triangularis; IFG:Op inferior frontal gyrus, pars opercularis, Or pars orbitalis; Tr pars triangularis; ifs inferior frontal sulcus; lf lateral fissure; MFG middle frontal gyrus; MTG middle temporal gyrus; PoG postcentral gyrus; PrG precentral gyrus; sts superior temporal sulcus; ts triangular sulcus.

**Fig. 2:**
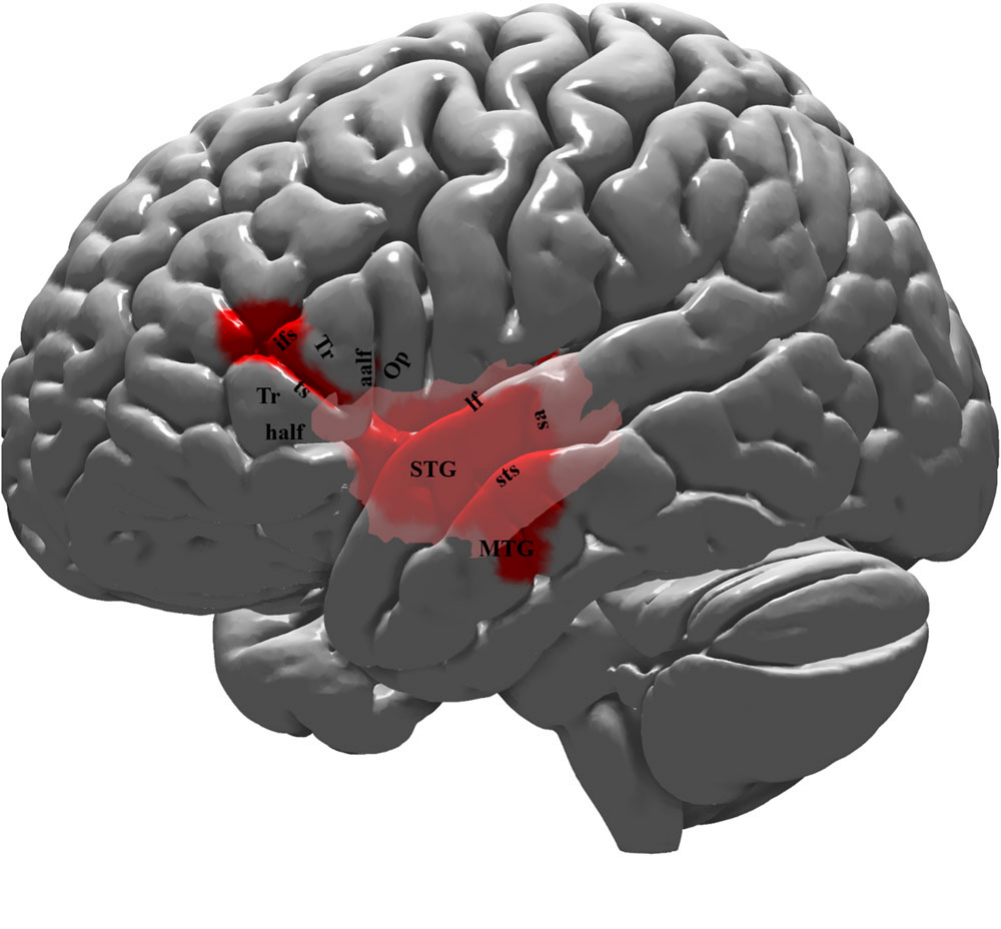
Lateral view of the left hemisphere lesion of the patient with damage to the ventral language network (patient AA). The cortical lesion is marked by the red color and occupies the superior and middle temporal gyri (STG and MTG) anterior to the sulcus acousticus (sa), as well as the pars triangularis (area 45) of the inferior frontal gyrus. Note that the posterior parts of the superior and middle temporal gyri (i.e. the classical Wernicke area) and also the cortex of the pars opercularis (area 44) are spared. Thus, the lesion is restricted to the ventral language stream. Subcortically, the lesion is represented by the pale pink color. The lesion of the patient was reconstructed in MNI stereotaxic space and projected on the standard average MNI brain^65,66^. Abbreviations: aalf ascending anterior ramus of the lateral fissure; half horizontal ascending ramus of the lateral fissure; ifs inferior frontal sulcus; lf lateral fissure; MTG middle temporal gyrus; Op pars opercularis (area 44); sa sulcus acousticus; STG superior temporal gyrus; sts superior temporal sulcus; Tr pars triangularis (area 45); ts triangular sulcus.

Patient AA is a 42-year-old man, examined 1.5 years after a left hemisphere CVA that affected the anterior parts of the superior and middle temporal gyri, as far posteriorly as the sulcus acousticus (see Figs. 1 and 2). Lesion analysis showed that the cortex of the temporal pole as well as the cortex posterior to the sulcus acousticus, i.e. the classic dorsal posterior temporal language region (Wernicke’s area), was completely spared. In addition, there was specific damage to the pars triangularis of the IFG where granular area 45 lies with complete sparing of the pars opercularis (dysgranular area 44) and no other damage in the frontal lobe (see Fig. 2). There was no damage to the occipital lobe or the adjacent occipito-temporal region. This is important because at times impairments related to the ventral language system, namely the anterior temporal cortical region, have been incorrectly attributed to damage of the multicomponent so-called inferior fronto-occipital fasciculus. Damage was also observed in the internal capsule, external capsule, parts of the basal ganglia, and the anterior insula. DTI analysis showed decreased fractional anisotropy (FA) in the TFexcF (Table 1) which is the white matter connection between the anterior lateral temporal region and the frontal cortex, and on the IFG specifically with the pars triangularis where area 45 of Broca’s region lies. Thus, the CVA lesion affected selectively the ventral anterior temporal language system.

**Table 1:** Presentation of the Fractional Anisotropy (FA) values of the white matter fasciculi reconstructed for the three patients and the control group.

	L TFexcF FA	L SLF III FA	L AF FA	L SLF II FA
AA	0.32580*	0.43999	0.49847**	0.42856
	*p* = 0.005	*p* = 0.059	*p* = 0.005	*p* = 0.074
	*r* = 0.886	*r* = 0.596	*r* = 0.886	*r* = 0.247
MM	0.43417	0.35495*	-	-
	*p* = 0.059	*P* = 0.003		
	*r* = 0.596	*r* = 0.886	-	-
TA	0.43237	0.38423*	0.41299*	-
	*p* = 0.508	*p* = 0.005	*p* = 0.005	
	*r* = −0.209	*r* = 0.886	*r* = 0.886	
Controls				
Mean	0.43713	0.44594	0.47733	0.43640
(SD)	(0.0187)	(0.0142)	(0.0155)	(0.0128)

	L IL F FA	L MdLF FA	L CB FA	
AA	0.46635**	0.47095**	0.45929	
	*p* = 0.005	*p* = 0.005	*p* = 0.093	
	*r* = −0.886	*r* = −0.886	*r* = 0.531	
MM	0.40857*	0.30334*	0.42891	
	*p* = 0.005	*p* = 0.005	*p* = 0.074	
	*R* = 0.886	*r* = 0.886	*r* = 0.564	
TA	0.41299*	0.38169*	0.46329	
	*p* = 0.005	*p* = 0.005	*p* = 0.139	
	*R* = 0.886	*r* = 0.886	*r* = 0.467	
Controls				
Mean	0.43582	0.42705	0.46456	
(SD)	(0.0092)	(0.0183)	(0.0449)	

Fractional Anisotropy (FA) values of the left hemisphere Temporo-Frontal extreme capsule Fasciculus (TFexcF), Superior Longitudinal Fasciculus branch II (SLF ΙΙ) and branch III (SLF ΙΙΙ), Arcuate Fasciculus (AF), Inferior Longitudinal Fasciculus (ILF), Middle Longitudinal Fasciculus (MdLF) and Cingulum Bundle (CB), which was used as a control tract are also presented in the left hemisphere (LH) for the three patients and the control group (*n* = 10), are presented. Mean FA and standard deviation (SD) of all white matter tracts included in the study are reported for the control group. (-) Reconstruction failure of the tracts because of the extensive damage in the white matter where they course resulting from the left CVA. *P*-values (*p*) as well as effect sizes (*r*) are calculated and presented for each participant and tract reconstruction. *Significantly lower FA values, as calculated with the use of the Wilcoxon signed rank test, with significance level set at 0.05. **Significantly higher FA values as calculated with the use of the Wilcoxon signed rank test, with significance level set at 0.05.

### Language and general neuropsychological performance

Patient AA’s speech was remarkably fluent and without paraphasic errors. His naming ability was intact and so was performance on the Boston Diagnostic Aphasia Examination (BDAE) subtests assessing repetition of aurally presented words and sentences as well as reading. Comprehension of words and sentences, as assessed by the BDAE, was preserved. Immediate and working auditory memory, as measured by the Digits Forward and Digits Backwards Tests, as well as immediate and working visuospatial memory, measured by the Corsi Forward and Backwards test, respectively, were within normal range (Table 2). Visuo-constructional ability (Taylor CF copy test) and visual memory (Taylor CF recall test) were intact and so was semantic knowledge and praxis. Finally, visuospatial scanning, processing speed, and mental flexibility/divided attention, as measured by the Trail Making Test A and B, were also preserved.

**Table 2:** Neuropsychological and Language performance of the three patients.

		Patients	
Task	AA	MM	TA
WC	16/16	15.5/16	14.5/16
SC	10/10	2/10	8/10
CM	5/6	0/6	4/6
ACi	25^th^%ile	<5^th^%ile^a^	<5^th^%ile^a^
WRp	5/5	2/5^a^	5/5
SRp	2/2	0/2^a^	1/2^a^
SS w/m	>50^th^%ile	24^th^%ile	<5^th^%ile^a^
CTP w/m	15^th^%ile	<5^th^%ile^1^	<5^th^%ile^a^
WRd	15/15	15/15	13/15^a^
SRd	42/43	43/43	15/43^a^
RC oral	2/3b^b^	2/3^b^	2/3^b^
RC silent	4/4	4/4	4/4
BNT	>50^th^%ile	10^th^%ile	20^th^%ile
PPVT-R	<5^th^%ile^a^	10^th^%ile	8^th^%ile
COWF-S	<5^th^%ile^a^	<5^th^%ile^a^	<5^th^%ile^a^
COWF-Ph	<5^th^%ile^a^	<5^th^%ile^a^	<5^th^%ile^a^
Corsi-F	11^th^%ile	66^th^%ile	<5^th^%ile^a^
Corsi-B	28^th^%ile	60^th^%ile	<5^th^%ile^a^
TaylorCFc	50^th^%ile	-	<5^th^%ile^a^
TaylorCFr	15^th^%ile	-	5^th^%ile^a^
DS-F	25^th^%ile	<5^th^%ile^a^	<5^th^%ile^a^
DS-B	25^th^%ile	<5^th^%ile^a^	<5^th^%ile^a^
PPT	15/15	-	13/15^a^
TMT-A	25^th^%ile	-	<5^th^%ile^a^
TMT-B	25^th^%ile	-	-
P-TU	53/55	-	-
I-H	19/20	-	-
I-F	16/20	-	-

Performance of the three patients in specific language and neuropsychological tests is presented.

*WC* word comprehension BDAE test, *SC* simple command comprehension BDAE test, *CM* complex material comprehension BDAE test, *ACi* auditory comprehension index; *WRp* word repetition BDAE test, *SRp* sentence repetition BDAE test, *SS w/m*: Stroke Story words/minute BDAE test, *CTP w/m* Cookie Theft Picture words/minute test, *WRd* word reading BDAE test, *SRd* sentence reading BDAE test, *RC* reading comprehension BDAE test, *Corsi-F* Corsi Block-tapping Task forward condition, *Corsi-B* Block-tapping Task backward condition, *Taylor CFc* Taylor complex figure copy, *Taylor CFr* Taylor complex figure recall, *PPVT-R* Peabody vocabulary test-revised, *COWF-S* semantic controlled oral word fluency, *COWF-Ph* phonemic controlled oral word fluency, *TMT-A* Trail Making Test part A, *TMT-B* Trail Making Test part B, *BNT* Boston Naming Test, *DS-F* Digit Span forward condition, *DS-BB* Digit Span backward condition, *P-TU* Pantomime of Tool Use test, *I-H* Imitation of Hand test; *I-F* Imitation of Fingers test; *PPT* pyramids and palm trees test.

^a^Impaired performance on the basis of normative data provided by the corresponding studies cited in text.

^b^Although the group that conducted the adaptation/standardization study did not provide percentiles for separate subscales, these scores were judged as being mildly impaired by an experienced neuropsychologist specialized in aphasia (D.K.).

However, his performance on the Peabody Picture Vocabulary Test (PPVT-R), a receptive vocabulary comprehension test, was significantly reduced. Further neuropsychological evaluation revealed significant difficulties in semantic fluency and phonemic fluency (COWF test) that may reflect a selective retrieval problem due to the failure of interaction between the pars triangularis (area 45) and the anterior semantic temporal region via the TFexcF. As shown in Table 2, AA’s neuropsychological testing is more extensive in comparison with that of the other two patients who had the classic posterior dorsal temporal lesions. This decision for further assessment of patient AA (case 1) was made in order to document and confirm his selective deficits, given his unique and extremely rare anatomical lesion that disrupted selectively the anterior temporo-frontal system via the extreme capsule fasciculus. The other two patients MM (case 2) and TA (case 3) were examined with the standard aphasia protocol of our clinic.

### Case 2 (MM)

**Fig. 3:**
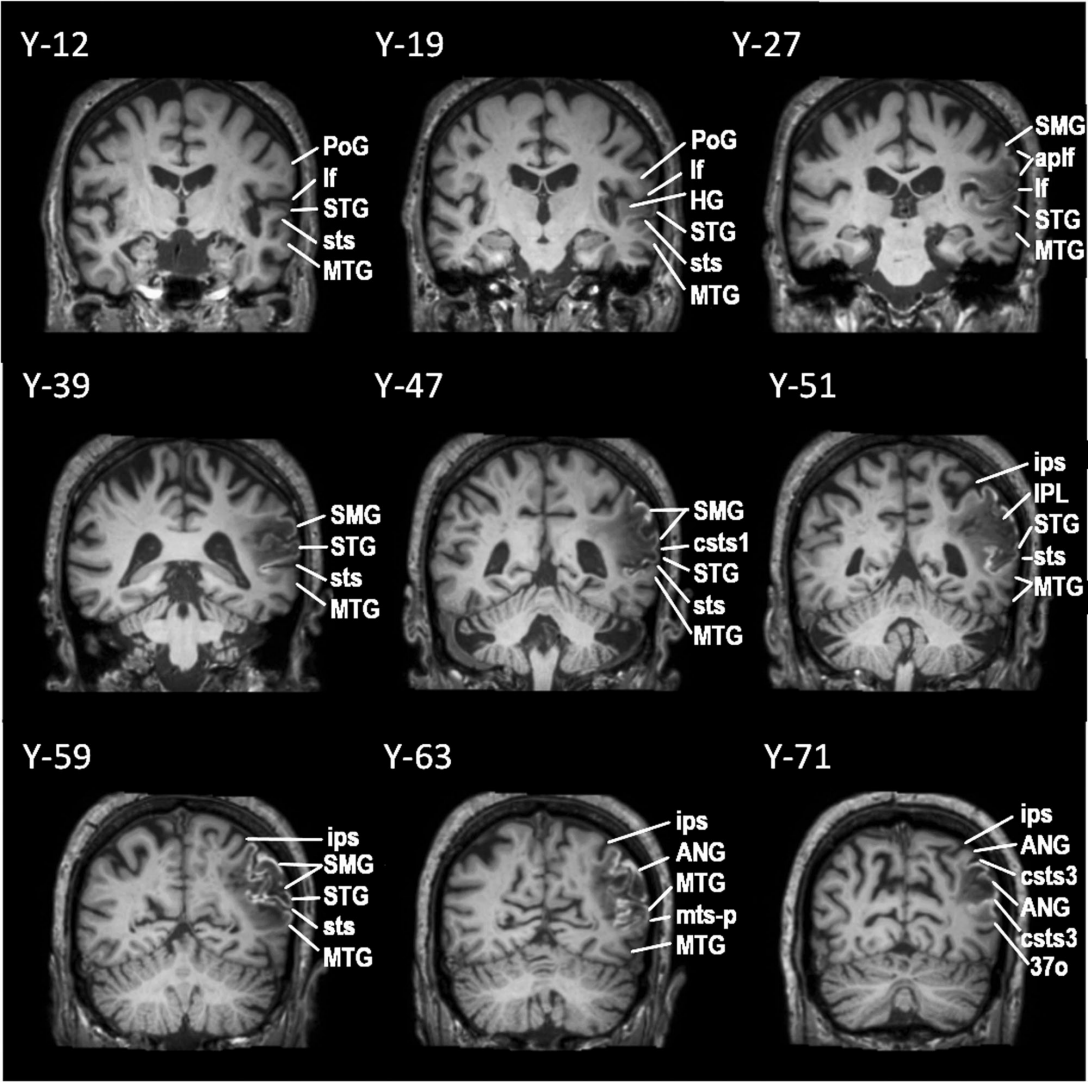
Successive coronal MRI images in MNI stereotaxic coordinates (Y) depicting the lesion of patient MM with damage to the dorsal posterior language region. The lesion first appears below the central sulcus, just posterior to the sulcus acousticus, at Y-12, where a small part of the STG is damaged. At approximately Y-19, there is damage to both the planum temporale, where HG lies and the lateral part of the STG, including both banks of the sts (involving the classic Wernicke region). At Y-27, there is complete damage to HG, planum temporale, the entire STG and both banks of sts. At this level, one observes damage to the parietal operculum and the anterior part of SMG (area PF). At Y-39, there is still damage to the entire STG, including both banks of the sts. The parietal operculum, and the ventral part of SMG are completely destroyed, as well as the underlying white matter, which affected fibers from both the SLF III and AF. Further posteriorly, at Y-47, the entire SMG, STG and both banks of sts, as well as the underlying white matter is destroyed. At Y-51, the lesion includes the entire STG and both banks of the sts. The lesion extends from the ips, down to the posterior temporal lobe. Thus, all of the SMG was damaged including the adjacent STG and the upper part of adjacent MTG. At Y-59, again there is damage in the SMG gyrus and the underlying white matter. The damage extends from the ips as far as the posteriormost part of the adjacent MTG. At Y-63, the lesion extends below the ips including the ANG and MTG, until the mts-p. Between Y-71 and Y-79, the lesion includes the ANG from the intraparietal sulcus as far ventral as the csts-3. Brain areas are topologically defined according to the atlas of the morphology of the human cerebral cortex in the MNI Stereotaxic Space^67^. ANG angular gyrus; aplf ascending posterior ramus of the lateral fissure; csts1 caudal superior temporal sulcus, branch 1; csts3 caudal superior temporal sulcus, branch 3; HG Heschl’s gyrus; IPL inferior parietal lobule; ips intraparietal sulcus; lf lateral fissure; MTG middle temporal gyrus; mts-p posterior middle temporal sulcus; PoG postcentral gyrus; SMG supramarginal gyrus; STG superior temporal gyrus; sts superior temporal sulcus.

**Fig. 4:**
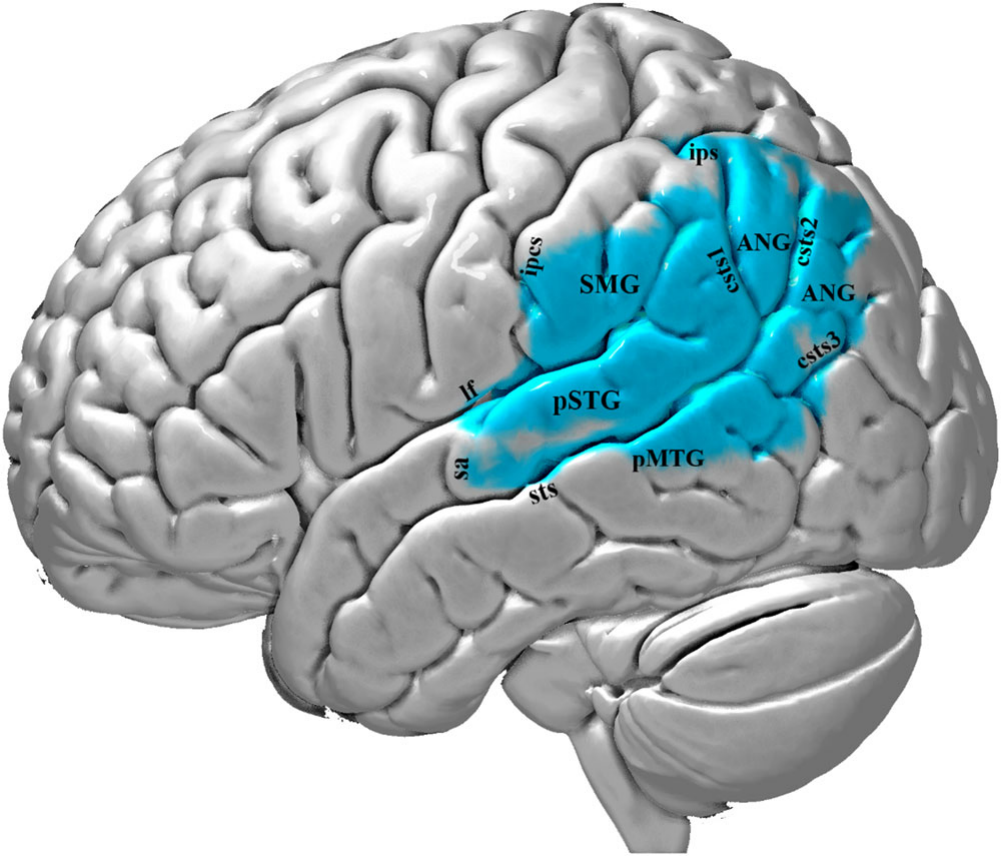
Lateral view of the left hemisphere lesion of patient MM with damage to the dorsal posterior language region. The area affected by the lesion is marked with the blue color. The lesion includes the posterior temporal region, i.e. clearly posterior to the level of the sulcus acousticus (sa). It also includes the Heschl’s gyrus (HG) region, which lies within the lateral fissure, and a part of the posterior middle temporal gyrus (pMTG). Thus, the posterior temporal region (Wernicke’s area) is damaged. The lesion extends to the supramarginal (SMG) and angular (ANG) gyri of the inferior parietal lobule. Thus, the lesion is restricted to the dorsal posterior language network, sparing all cortical areas associated with the ventral language network, i.e. the anterior to intermediate temporal areas and their connections via the TFexcF to area 45 on the IFG. The lesion was reconstructed in MNI stereotaxic space and projected on the standard average MNI brain^65,66^. Abbreviations: ANG angular gyrus; csts1 caudal superior temporal sulcus, first segment; csts2 caudal superior temporal sulcus, second segment (angular sulcus); csts3 caudal superior temporal sulcus, third segment (anterior occipital sulcus); ipcs inferior postcentral sulcus; ips intraparietal sulcus; lf lateral fissure; pMTG posterior middle temporal gyrus; pSTG posterior superior temporal gyrus; sa sulcus acousticus; SMG supramarginal gyrus; sts superior temporal sulcus.

MM is a 72-year-old man, who was examined 7 months after a left hemisphere CVA. The lesion involved the posterior parts of the superior and middle temporal gyri, the adjacent posterior insula (extending to the external capsule) and a large part of the inferior parietal lobule, i.e. parts of the angular gyrus (ANG) and most of the supramarginal gyrus (SMG), sparing its anterior dorsal part (see Figs. 3 and 4). DTI data analysis showed decreased FA in the third branch of the superior longitudinal fasciculus (SLF III) which connects the supramarginal gyrus with the IFG, and the arcuate fasciculus (AF) which connects the dorsal posterior temporal region (Wernicke’s area) with the IFG. Moreover, the second branch of the superior longitudinal fasciculus (SLF II) could not be reconstructed because of the severe damage to the relevant white matter (see Methods section). SLF II, originating from the angular gyrus (BA 39), consists of a ventral branch which targets area 45 (pars triangularis) on the IFG, and a dorsal branch which targets the posterior region of the superior and middle frontal gyri where BA 8 lies^11–15,27^. Thus, in this patient, there was severe damage to the posterior dorsal language network and the three pathways that connect this network with the IFG. Importantly, there was no damage to the ventral anterior temporal language network and the TFexcF that connects the anterior temporal region with the IFG (Table 1), although a significant FA decrease was observed in the ILF and MdLF because of the lesion in the posterior inferior temporal areas (see Fig. 3). Thus, this patient had a classic lesion in the dorsal posterior language network, impairing the posterior dorsal temporal region (Wernicke’s area) and the adjacent inferior parietal region (the angular and supramarginal gyri), as well as the connections linking these areas with Broca’s speech production region on the IFG via the AF (from dorsal posterior temporal region), the SLF II (from the angular gyrus) and the SLF III (from the supramarginal gyrus).

The speech of this patient included phonemic paraphasias, neologisms and, overall, reduced information transmission, as well as word finding difficulties and use of circumlocutions. His naming performance, despite the occurrence of some phonemic paraphasias and neologisms, was in the normal range. Repetition of words and sentences was impaired. Orally presented sentence and complex material comprehension was decreased, as shown by the significantly decreased auditory comprehension index (see Table 2), but single word comprehension was largely spared, as could be seen in the PPVT-R as well as the BDAE word comprehension test (Table 2). Reading out loud and reading comprehension were spared. Performance on measures of verbal working memory was significantly impaired (Table 2). Visual immediate and working memory were spared. Thus, this patient exhibited impairments typical of damage to the dorsal posterior language system.

### Case 3 (TA)

**Fig. 5:**
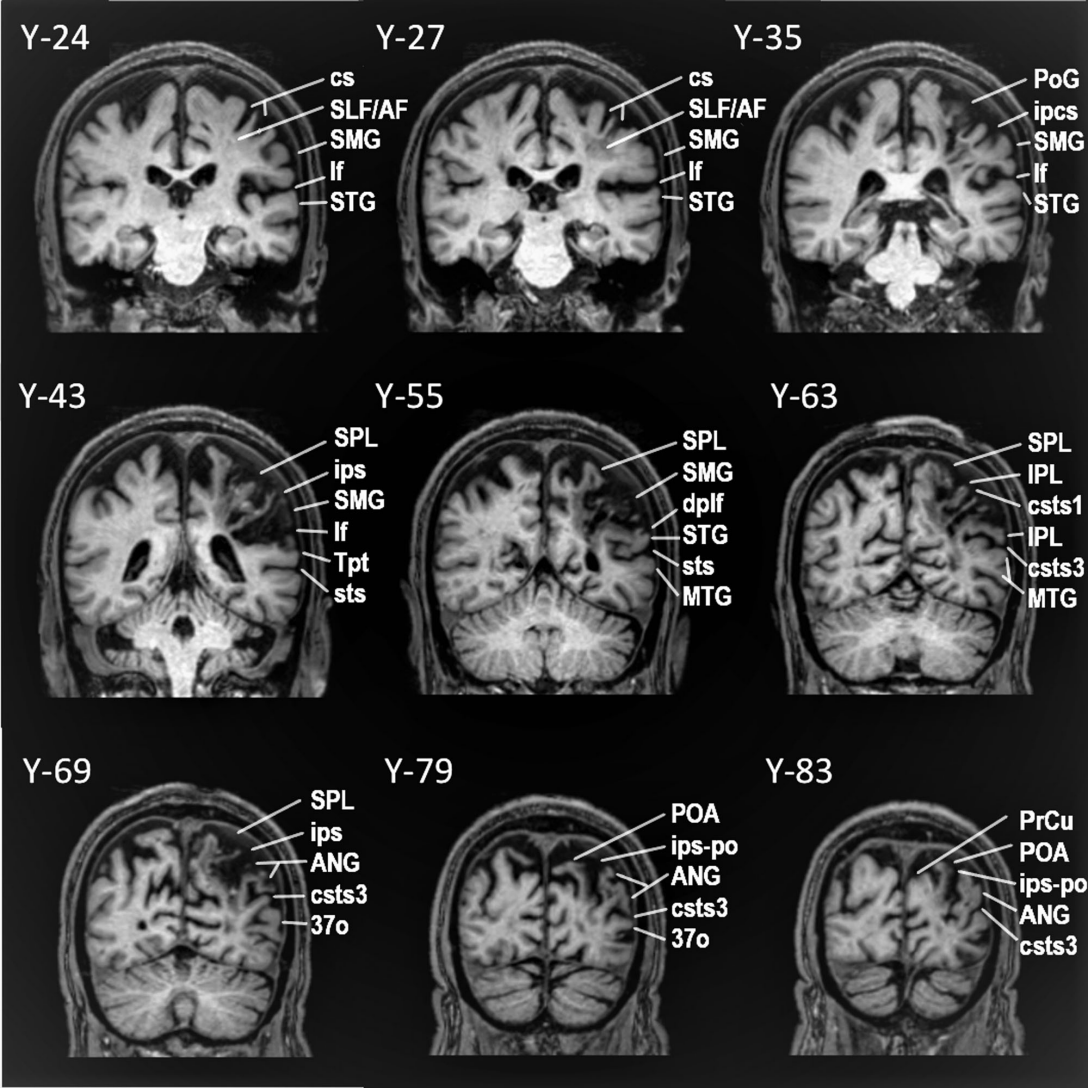
Successive coronal MRI images in MNI stereotaxic coordinates (Y) depicting the lesion of patient TA with damage to the dorsal posterior language region. The lesion starts posterior to the central sulcus (cs) and after Y-24. At Y-27, the white matter below SMG and postcentral gyrus areas 2, 1, 3b is damaged affecting SLF III fibers (originating from the SMG), SLF II fibers (originating from ANG), and AF fibers (originating from Tpt. At Y 31, a small area of anterior SMG is added to the white matter lesion. At Y-35 and Y-39, the lesion includes SMG and the white matter below. At Y-43, the whole SMG, the white matter below and a small part of area Tpt, that is the caudal STG, is damaged, leaving primary acoustic areas on Heschl’s gyrus intact. Further posteriorly, at Y-47, -51, -55 as well as -59, damage continues to include the whole SMG and SPL (Superior Parietal Lobe), occupying a larger part of STG (see Y-55). At Y-63, superior and inferior parietal lobule areas, including areas PFm and PG, as well as the white matter below, until csts3 are affected. At Y-67 till Y-71, the superior and inferior parietal lobule areas, including ANG, as well as the underlying white matter until the area just below csts2, are lesioned (see Y-69). At Y-75, Y-79, and Y-83, the precuneus, superior and inferior parietal lobule areas until csts3 are affected, while at Y-83, the lesion includes the areas of POA and the whole ANG until csts3. The lesion continues further posteriorly to parieto-occipital areas, affecting even occipital areas such as area 19, in Y-98, where the lesion ends. Brain areas are topologically defined according to the atlas of the morphology of the human cerebral cortex in the MNI Stereotaxic Space^67^. 3a: area 3a; 37o: lateral occipitoparietal area 37; AF Arcuate Fasciculus; ANG angular gyrus; cs central sulcus; csts1, csts3 caudal superior temporal sulcus, 1st and 3rd ramus; IPL inferior parietal lobule; ipcs inferior postcentral sulcus; ips intraparietal sulcus; ips-po intraparietal sulcus, paroccipital part; lf lateral fissure; MTG middle temporal gyrus; POA parieto-occipital arcus; PoG postcentral gyrus; PrCu precuneus; SLFII/III superior longitudinal fasciculus, subdivision II/III; SMG supramarginal gyrus; SPL superior parietal lobule; STG superior temporal gyrus; sts superior temporal sulcus; Tpt caudal superior temporal cortical region.

**Fig. 6:**
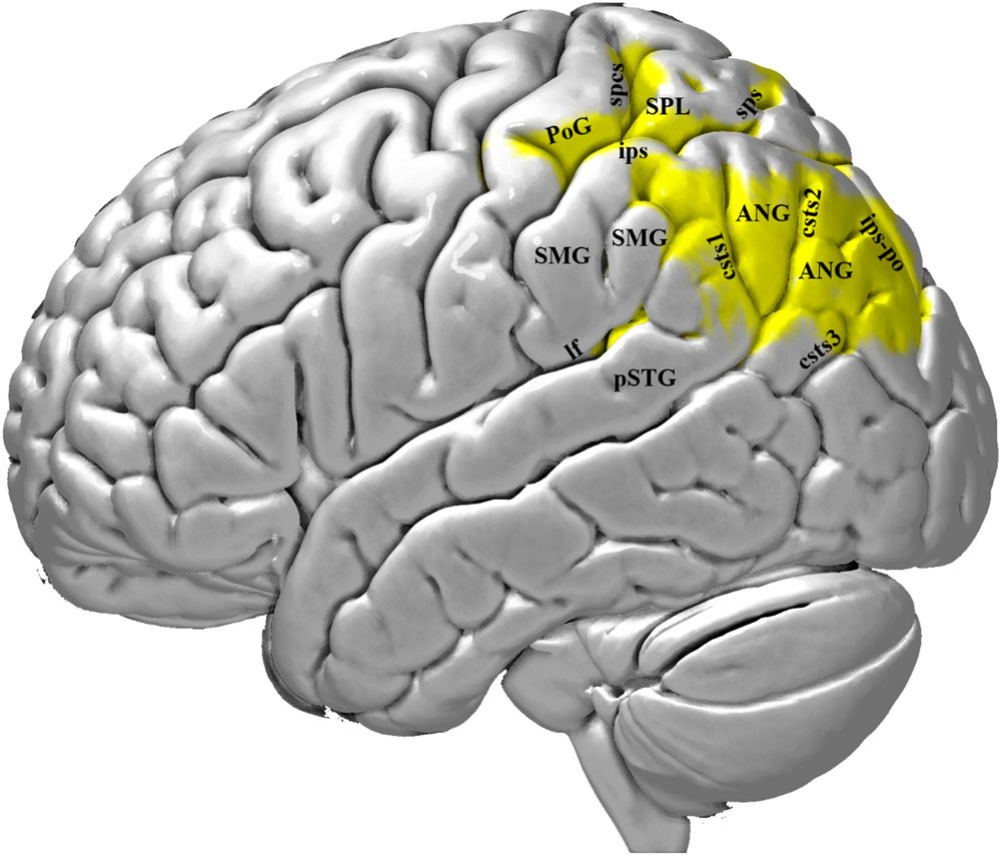
Lateral view of the left hemisphere lesion of patient TA. The area invaded by the lesion is marked with the yellow color and involves mostly the parietal lobe, including some adjacent posterior temporal and occipital areas. Thus, the lesion is restricted to the dorsal posterior language network, sparing all cortical areas associated with the ventral language network, such as the pars triangularis (area 45) of the inferior frontal gyrus and the anterior temporal areas. The lesion was reconstructed in MNI stereotaxic space and projected on the standard average MNI brain^65,66^. Abbreviations: ANG angular gyrus; csts1 caudal superior temporal sulcus, first segment; csts2 caudal superior temporal sulcus, second segment (angular sulcus); csts3 caudal superior temporal sulcus, third segment (anterior occipital sulcus); ips intraparietal sulcus; ips-po intraparietal sulcus, paroccipital segment; lf lateral fissure; pSTG posterior superior temporal gyrus; PoG postcentral gyrus; SMG supramarginal gyrus; spcs superior postcentral sulcus; sps superior parietal sulcus.

TA is a 72-year-old man who suffered a CVA in the left hemisphere a year prior to the examination. The lesion included a small portion of the most posterior part of the superior temporal gyrus (STG), and parts of the inferior parietal lobule, such as the supramarginal (SMG) and angular (ANG) gyri (see Figs. 5 and 6). DTI tractography demonstrated decreased FA in the SLF III and the AF, and reconstruction of the SLF II failed because of the extent of the damage in the relevant white matter. Moreover, there was significant FA decrease in the ILF and MdLF, compared to the healthy controls, as would be expected given the partial damage to the posterior terminations of these tracts as a result of the location of this patient’s lesion (see Fig. 5). In contrast to case MM, Heschl’s gyrus and surrounding areas of the posterior STG were spared. However, the lesion of case TA extended further posteriorly to involve a larger part of the ANG and adjacent parieto-occipital cortex.

**Fig. 7:**
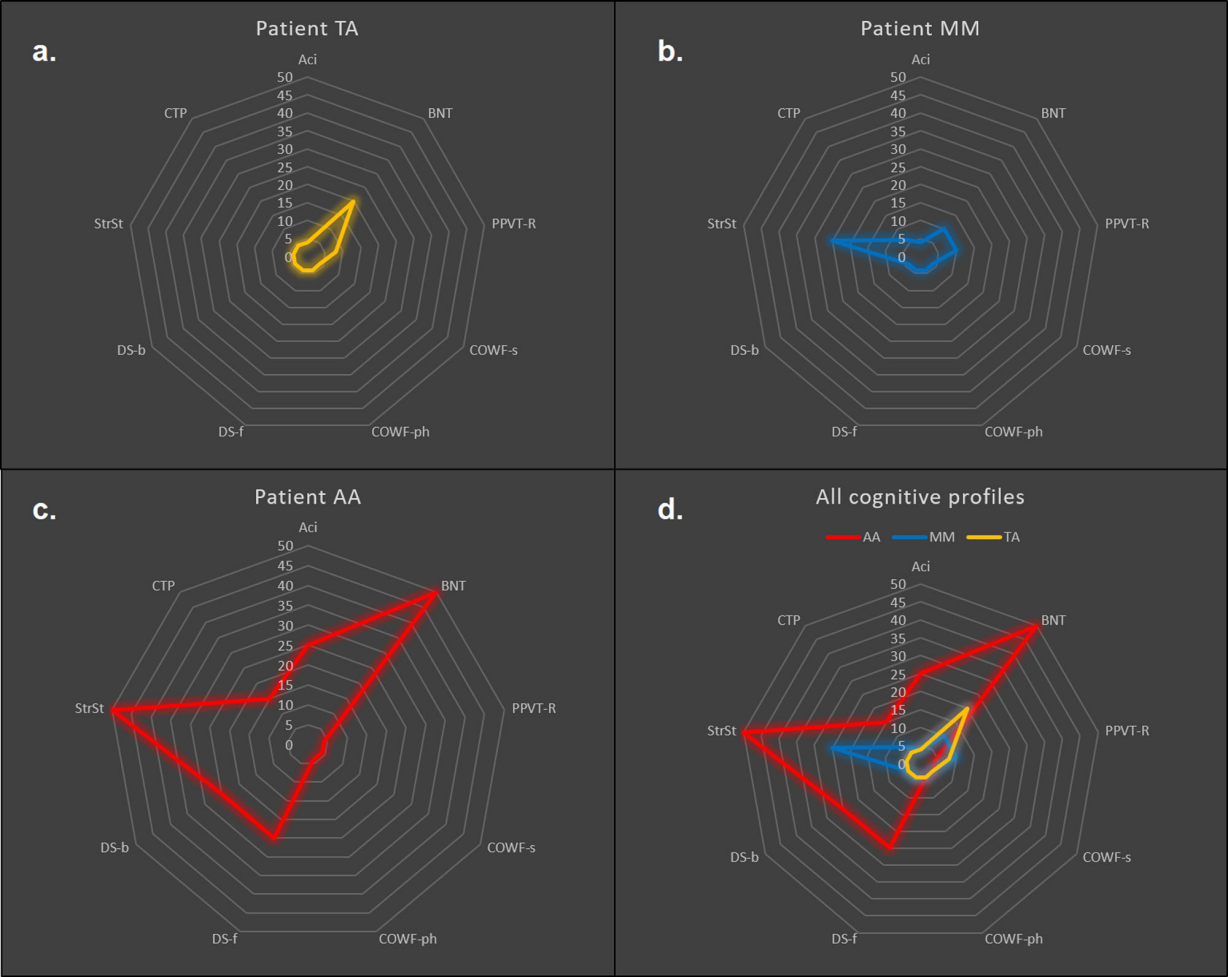
Individual language and cognitive profiles for the three patients. The vertical axis shows percentiles up to the 50th in all graphs. For each neuropsychological test, raw scores were transformed into percentiles on the basis of the corresponding normative studies (cited in Methods). For speech rate in the stroke story and cookie theft picture description, percentiles were calculated based on a group of healthy participants sampled from the project “Investigation of cortical surface patterns and their relation with speech metrics and performance in neuropsychological assessment in healthy participants” carried out at Aeginition Hospital in Athens, School of Medicine, Greece (research protocol approval ID: ΩΟΞΛ46Ψ98N2-7PN, July 2017). In **a**, performance of patient TA is represented with yellow. In **b**, performance of patient MM is represented with blue and in (**c**), performance of patient AA is represented with red. In (**d**), all profiles are shown for comparison. As one can observe in (**c**), the differential performance of AA in specific domains is manifested, i.e. impaired performance in tests involving selective retrieval vs. non-impaired performance in tests measuring other cognitive processes (points within the inner polygon correspond to impaired performance, below the 5th percentile). In (**d**), the combined profiles illustrate the differences in the performance of AA (red polygon) compared to MM (blue polygon) and TA (yellow polygon). While AA shows generally preserved language and cognitive abilities, his scores in measures requiring selective retrieval (i.e. PPVT-R, COWF-s, COWF-Ph) is significantly decreased, manifesting lower performance compared to MM and TA. However, the two posterior CVA patients despite their generally decreased performance, scored higher than AA in procedures requiring selective retrieval. Repetition cannot be visualized in the above graphs, because Tsapkini and colleagues^28^ did not report percentiles for the BDAE repetition subscale alone. Nevertheless, we clarify in the text that repetition skills of AA were intact in contrast to the other two patients. ACi auditory comprehension index; BNT Boston Naming Test; PPVT-R Peabody vocabulary test-revised; COWF-s semantic subscale of Controlled Oral Word Fluency; COWF-ph phonemic subscale of Controlled Oral Word Fluency; DS-f Digit Span forward condition; DS-b Digit Span backward condition; StrSt Stroke Story speech rate (words/minute); CTP Cookie Theft Picture speech rate (words/minute).

Although patient TA could communicate information adequately, his speech included phonemic paraphasias, a few syntactic errors, word finding difficulties with pauses, and characteristic multiple attempts to articulate words correctly. His naming abilities as measured by the BNT were within the normal range. The overall auditory comprehension index was low, mainly due to reduced scores in the orally presented sentence and complex material comprehension subscales. However, receptive vocabulary, as measured by PPVT-R, was within the normal range. He had difficulty in reading sentences aloud, with regular occurrence of phonemic paraphasias, and word omissions. Comprehension of written sentences was intact. Word repetition was spared, but difficulty in sentence repetition was observed. Difficulty was also observed on measures of auditory immediate and working memory. His visuo-constructive abilities were decreased as seen in his performance on the Taylor Complex Figure test. Visual attention and working memory deficits were also observed. Psychomotor speed, visuospatial scanning and divided attention were significantly impaired, as measured by the Trail Making Test. Performance on measures of word fluency was decreased. Finally, performance on a word-object knowledge test (PPT) was slightly reduced, possibly due to his visuospatial difficulties (Table 2). Individual cognitive profiles are shown in Fig. 7.

As presented above, in patients MM and TA who had a lesion affecting the dorsal posterior superior temporal cortex and adjacent cortex in the nearby inferior parietal lobule (Figs. 3–6), it is clear that the dorsal posterior language network was severely damaged. Additionally, the connectivity of the posterior temporal and inferior parietal lobule with frontal cortical regions was impaired: the SLF II and AF could not be reconstructed in the case of MM because of severe damage to the relevant white matter, including decreased FA value in SLF III (Table 1), while in the case of TA, SLF II could not be reconstructed, and SLF III and AF showed decreased FA. On the contrary, in both of these patients, the TFexcF was not damaged, permitting interaction between the anterior temporal region and area 45. Thus, in both patients, language performance that depends largely on connections between the anterior lateral temporal cortex and the pars triangularis (area 45) of the inferior frontal region (and other lateral frontal areas) via the TFexcF was still functional. Furthermore, the precentral motor and postcentral somatosensory areas were intact in these patients and, therefore, communication between area 44 on the pars opercularis and the adjacent orofacial premotor and motor region on the precentral gyrus was intact. Thus, it is clear that the dorsal posterior language network in these two patients was severely damaged.

On the other hand, patient AA (Figs. 1 and 2), manifested lesion in the anterior to intermediate temporal areas, the IFG, and the white matter connecting them (i.e. the TFexcF). However, temporal areas posterior to the sulcus acousticus, parietal areas as well as all the dorsal white matter tracts (SLF II/III and AF) remained intact. Thus, in patient AA the lesion was restricted to the ventral language system.

Specifically, although there was damage to the pars triangularis (area 45) of the IFG, there was no damage to the pars opercularis (area 44) of the IFG, i.e. the region electrical stimulation of which leads to pure speech arrest, or the ventral precentral motor region stimulation of which leads to vocalization and interference with speech^23^ (see Supplementary Fig. 1). This is the first major finding from the examination of this case, and it raises the following question: what might be the specific functional role of area 45, i.e. the anterior granular component of Broca’s region?

The neuropsychological performance of the three patients, as can been seen in Fig. 7, showed that AA’s language/cognitive profile is distinct compared to the profiles of the other two patients. If the scores are examined individually, there are some observed overlaps between the three cases (e.g., all three patients score below the 5th percentile in the fluency scales). However, an overall assessment of the cognitive skills of the patients, focusing on identification of impairment in one or more tasks in relation to performance on other tasks, is much more informative, revealing differences in the profiles, rather than overlaps or discrepancies in performance on separate tests. In summary, these patients are considered on the basis of both their deficits and preserved abilities. In this manner, the contrast between patient AA compared to the other two patients with the damage in the classic dorsal posterior temporal lobe network becomes very informative regarding the role of the dorsal and ventral language networks. Differential performance was observed in specific domains that highlights the selectivity of the impairment in case AA in comparison with the two cases who had the classic posterior temporo-parietal lesions (cases MM and TA). These differences are listed here in points a to d.

- Differences in word comprehension, as measured by the PPVT-R vs the BDAE word comprehension subtest: Patients MM and TA demonstrated the typical aphasic profile, showing mildly reduced performance in the BDAE word comprehension subscale, and overall impaired scores in the BDAE comprehension scale, and low scores, although within the normal range, in the PPVT-R scores. In contrast, patient AA exhibited perfect performance in the classic word comprehension subscale and overall non-impaired performance in the comprehension scale of the Boston Aphasia Examination Battery (BDAE), see auditory comprehension index (ACi) in Table 2 and Fig. 7. However, his performance in the PPVT-R was significantly reduced. Taking into consideration the fact that BDAE is a battery designed for clinical assessment of patients with aphasia, in which the scores of non-aphasic individuals manifest ceiling effects^28^, whereas the PPVT-R scores show much greater variance in the healthy population, and the test is generally considered to be more demanding, and therefore more sensitive to subtle deficits^29^, it is clear that AA’s neuropsychological profile does not coincide with the typical fluent aphasia, but demonstrates a specific deficit concerning retrieval of phonemic/semantic representations under demanding conditions.
- Differences in sentence comprehension, as measured by the sentence and complex material comprehension subtests of the Boston Aphasia Examination Battery (BDAE): AA’s performance was unremarkable, while MM’s and TA’s performance was reduced, leading to the conclusion that AA’s deficit was not related to a compromised semantic mechanism.
- Differences in sentence repetition, as measured by the BDAE sentence repetition subtest. The performance of patient AA was unremarkable, while the performance of the patients MM and TA was reduced. The observed normal sentence repetition performance of patient AA can be attributed to the intact dorsal posterior temporal language system. The latter interpretive schema is further supported by findings regarding immediate and working auditory memory, as measured by the Digits Forwards and Digits Backwards tests in which the performance of patient AA was within the normal range, while the performance of patients MM and TA was impaired, as expected.
- Finally, while patient AA showed absence of errors during oral or written speech, patients MM and TA presented with frequent errors and neologisms. The absence in patient AA, but the presence in patients TA and MM, of phonemic errors/neologisms during speech is in accordance with the interpretation made above that AA’s overall cognitive/language profile cannot be associated with a general semantic breakdown, but it reflects a selective impairment in controlled retrieval from the semantic system (see Fig. 7).

Despite fluent and phonemically errorless speech, patient AA presented with a specific cognitive deficit. His performance on the PPVT-R test that assesses comprehension of individual words of increasing difficulty and is an excellent index of scholastic aptitude and general verbal ability was impaired (Table 2). However, he was able to understand basic words, such as high frequency concrete nouns (e.g., bear) and words denoting letters or colors, as shown by his intact performance on the word comprehension subtest of the BDAE. He was also able to follow oral instructions, co-operate, and respond appropriately in the interview session, as well as to understand sentences in the BDAE sentence comprehension subtest (Table 2). What is the difference in language comprehension as measured by the above two distinct single word comprehension tests? In both the BDAE word comprehension subtest and the PPVT-R, the examinee is asked to indicate, on a stimulus plate, which one of four drawings corresponds to a spoken word. In the BDAE word comprehension subtest, this word is a high frequency noun, letter, number, color, or part of the body, but in the PPVT-R this word can be a concrete or abstract noun, a verb or adjective of increasingly lower imageability and frequency, and the patient is asked to match this word with scenes of potentially related objects, actions, sceneries or event situations of increasing difficulty. Abstract words have more complex semantic representations than concrete words and more variable meanings that change with context^30^. Thus, besides vocabulary knowledge, on the PPVT-R test, the participant must selectively retrieve the possible meanings of each word and must consider the possible interpretations of each drawing in order to decide what the optimum choice might be. This process of selecting among a set of relevant alternatives based on certain criteria involves active controlled selective retrieval of information from the relevant semantic networks. Selective retrieval is also required in order to retrieve from memory words that begin with a certain letter, or words that belong to a specific category, as is the case for the phonemic and category fluency tests (COWF, Table 2), i.e. tests in which patient AA showed significantly decreased performance. This active selective memory retrieval process that permits selection between competing stimuli that could have appeared in different contexts and relations with other stimuli has been defined as an executive control process that depends critically on ventrolateral granular area 45 and its connections^24^. For active controlled retrieval of verbal/semantic information from lateral temporal cortex, the TFexcF would be the critical pathway^24^ since it links the anterior lateral temporal region with area 45 of the IFG^11,12^, providing area 45 with access to auditory (anterior STG) and multisensory information (anterior MTG), which in the language dominant hemisphere are known to subserve verbal semantic processing^31–34^.

To our knowledge, AA is the first case where a lesion selectively affects the pars triangularis (area 45), but not pars opercularis (area 44; see Supplementary Fig. 1), both of which are parts of IFG, usually damaged together after a CVA. As described above, AA’s lesion, which is restricted within the ventral language system, is related with a specific difficulty in active selective controlled retrieval of lexico-semantic information. This finding might provide a possible explanation of what cognitive function was disrupted when Penfield observed these mild unspecified naming disturbances during the electrical stimulation of area 45.

There is now considerable neuroimaging evidence regarding the role of area 45 in active selective retrieval processes and this fundamental role subserves the selective retrieval of verbal information in the language-dominant hemisphere^24^. In the right hemisphere, it is involved in the selective retrieval for nonverbal visual and auditory information^35–37^. The role of the ATL in language processing had previously been noted in primary progressive aphasia (PPA) and semantic dementia patients who often have ATL atrophy^2,6,7^. A recent study by Mesulam and colleagues^4^ of PPA patients demonstrated a dissociation between the neural substrates of single word comprehension measured by the PPVT-R test that involved the left anterior temporal region, and the neural substrates of sentence comprehension which involved Wernicke’s area in posterior temporal cortex and adjacent inferior parietal lobule areas, as well as their connections to frontal areas.

Another finding was that the speech output of the two patients with damage to the dorsal posterior language system was characterized by phonemic paraphasias, and, in the case of patient MM, also neologisms. Here again recent improvements in our understanding of the anatomical connectivity have provided major insights that relate to the present findings^11–15^. It has been shown that area 44 on the pars opercularis of the IFG receives auditory information from the posterior temporal region (Wernicke’s region) via the AF and high-level somatosensory information from the SMG via the SLF III. Such processing is critical for the planning of speech output and transfer of information to the orofacial precentral motor region for speech articulation. Thus, area 44 has reciprocal communication with the above areas that set the ground for a constant auditory (via AF) and somatic sensory (via SLF III) interaction as we speak. Disturbance of the above circuits would result in defective auditory and somatic sensory information related to word production, resulting in multiple corrective attempts, phonemic paraphasias, or even jargon speech. Furthermore, it has been shown that lesions in the left posterior STG or left SMG, as well as lesions affecting the AF (i.e. the pathway that links the dorsal posterior temporal gyrus with frontal cortex) or SLF III (the pathway that links SMG with frontal cortex), result in phonemic paraphasias^33,37–40^.

MM’s speech was also characterized by neologisms and, sometimes, unsuccessful attempts to correct them. Neologisms, like phonemic paraphasias, are considered the result of a breakdown in phonological encoding^41^, and evidence suggests that both are attributed to the same mechanism with differing degrees of breakdown severity^42^. Furthermore, both TA and MM presented with sentence comprehension impairments (see Τable 2), but were capable of understanding single words. Thus, both patients were able to transform phonological information to lexical representations and have access to meaning. In the case of MM, since his entire left posterior temporal region (including Heschl’s gyrus) was damaged, perception and early stages of phonological processing could be mediated by his intact right primary auditory cortical region. Note that, in both TA and MM, the left anterior temporal region that is related to lexical semantic knowledge^35^ was spared, and, therefore, access to meaning could be achieved through these cortical areas, and even through areas of the right hemisphere, which is also capable of some single word comprehension^43,44^.

By contrast, successful comprehension of the meaning of complex sentences requires the ability to maintain audtory representations for sufficient time to allow for their combination into meaningful sentences^45^. This suggests an important role for auditory short-term memory, which in the case of both patients MM and TA was decreased. In a group study of 210 stroke patients, auditory short-term memory and speech comprehension have been found to co-vary and, also, to share a common substrate, the left posterior STG^46^.

## Conclusions

Τhe detailed examination of the performance of these three patients on a broad spectrum of language and other cognitive tasks supports the dual stream hypothesis for language, and further highlights the importance of the ventral language system with regard to selective controlled retrieval, lexico-semantic processing and language comprehension^4,18–20^. Additionally, the present results underline the involvement of the dorsal posterior language system in phonological and somatosensory processing, sentence and complex material comprehension, as well as auditory immediate and working memory.

Finally, to the best of our knowledge, this is the first time that area 45, i.e. pars triangularis of the IFG, is related to a specific cognitive process through a selective lesion that spares the adjacent area 44 (pars opercularis). Note that areas 44 and 45 together comprise the classical Broca’s area.

Limitations of the present study are the relatively low number of directions and b-values which were used in the DTI data acquisition. Moreover, better distortion and correction algorithms would help to provide higher levels of accuracy. Finally, this study, which focused on the examination of CVA patients in the chronic phase (AA: 1.5 years post stroke, MM: 7 months, TA: 1 year) cannot address anatomical issues prior to the brain insult or potential functional plasticity as a coping mechanism.

## Methods

### Participants

Three right-handed, chronic patients after a single left hemisphere middle cerebral artery CVA were included in the study. Apart from the language impairments reported in the present article, there were no other neurological or psychiatric conditions. One of the patients suffered an anterior lateral temporal lesion affecting a major component of the ventral pathway for language, while completely sparing the posterior temporo-parietal network for language. The other two patients had a posterior lesion that impaired the posterior temporo-parietal language network, while sparing the anterior lateral temporal system. Additionally, a control group of 10 right-handed male adults, with the absence of any neurological or psychiatric disease was used. All participants underwent anatomical MRI examination and the patients were also tested for language and other cognitive functions. Demographic data of the three patients and the control group are presented in Table 3.

**Table 3:** Demographic data of the three patients and the control group.

Patients							Controls (*n* = 10)			
ID	Gender	Handedness	YoE	Age	TPO	ST	Gender	Handedness	YoE mean (SD)	Age mean (SD)
ΑΑ	M	RH	17	42	17	5				
ΜΜ	M	RH	12	62	12	5				
TA	M	RH	12	73	12	3	10 M	10 RH	14 (3.26)	54.7(9.3)

Demographic data of the three patients and the control group (*n* = 10) are presented. *YoE* years of education, *Age* age at examination, *TPO* time post onset of CVA in months, *ST* Speech Therapy duration in months, *M* Male, *RH* Right-Handed.

All healthy control participants were sampled from the project “Investigation of cortical surface patterns and their relation with speech metrics and performance in neuropsychological assessment in healthy participants” conducted at Aeginition Hospital in the Athens School of Medicine, Greece (research protocol approval ID: ΩOΞΛ46Ψ8N2−7PN, July 2017). The data of the patients were derived from the project “Investigation of common anatomical substrate of linguistic and non-linguistic cognitive deficits in post-stroke aphasia”, conducted at Aeginition Hospital in the Athens School of Medicine, Greece (research protocol approval ID: ΩΣ3Ξ46Ψ8Ν2-00Φ, July 2017). Informed consent was obtained from all participants according to the guidelines of the Ethics Committee of the Aeginition Hospital, National and Kapodistrian University of Athens, Greece. To our knowledge, no bias that may in any way impact the results has taken place during the design, execution, analysis or interpretation of the data of this study.

### Neuropsychological evaluation

For the examination of language performance, the oral expression, comprehension, repetition, and reading subtests of the Greek version of the Boston Diagnostic Aphasia Examination – Short Form (BDAE-SF)^28^ were used. Speech fluency was measured by the speech rate (SR) in two BDAE-SF tasks: (a) the Stroke Story (SS) test which requires the patient to describe orally his/her stroke incident and (b) the Cookie Theft Picture (CTP) that requires the description of a picture depicting a cookie theft event. SR was calculated as the total number of words divided by the total duration of speech, in the form of words/minute^47^. Naming was measured with a Greek version of the Boston Naming Test (BNT)^29^. Verbal comprehension was assessed with the word comprehension subtest of the BDAE Short Form and with the Greek version of the Peabody Picture Vocabulary Test-Revised (PPVT-R), a measure of receptive vocabulary^29^. Further neuropsychological testing included the Controlled Oral Word Fluency (COWF) test^48^, the Trail Making test^49,50^, the Pyramids and Palm Trees test (PPT)^51^, the Taylor Complex Figure (CF) test^52^, the Imitation of Gestures test^53^, the Pantomime of Tool Use test^54^, a digit span task^55^ with two conditions forward and backward and the Corsi Block Tapping task^56^. The results of the neuropsychological evaluation are listed in Table 2. Patients’ neuropsychological testing was conducted within the framework of standard clinical assessment by a neuropsychologist blind to the aim of the study.

### Magnetic resonance imaging acquisition

The MRI brain scans (whole brain scans) of the three patients and the control group were performed on a Philips 3.0 T MRI system (Achieva TX, Best, The Netherlands) equipped with an eight channel Sense-head coil. The Imaging Protocol included a 1 mm isotropic high resolution 3DT1-weighted sequence (time of repetition, TR) = 9.9 ms, echo time (TE) = 3.7 ms, flip angle= 70, voxel-size = 1 × 1 × 1 mm, matrix size = 244 × 240), an axial single shot, spin echo, echo planar diffusion tensor imaging (DTI) sequence (30 diffusion encoding directions, TR: 7299 ms, TE: 68 ms, flip angle: 900, acquisition voxel size: 2 × 2 × 2 mm, sensitivity encoding reduction factor of 2, two b factors with 0 s/mm2 (low b), and 1000 s/mm^2^ (high b)). All images were visually examined by an MRI physicist to identify possible artifacts and by a neuroradiologist to exclude subjects with unforeseen findings.

### Diffusion tensor imaging (DTI) analysis

The DTI image processing and the tract reconstructions were performed with Brainance DTI Suite (Advantis Medical Imaging, Eindhoven, The Netherlands) which uses a robust deterministic algorithm able to reconstruct kissing and crossing fibers^57^. Motion and eddy-current correction of DTI data were implemented via the registration tool available in the scanner^58,59^ and co-registration took place before further analysis with the use of Brainance DTI suite. In order to reconstruct the selected white matter tracts, region of interest (ROI) tractography was used according to the protocols described below. Step size was set to 1, and length threshold to 200 mm. Angle and fractional anisotropy thresholds were set at 35° and 0.20, respectively, for the reconstruction of the AF, SLF II, SLF III, TFexcF, while 27° angle and 0.15 and fractional anisotropy thresholds were used in the case of MdLF, ILF and cingulum bundle. The mean fractional anisotropy values of the pathways were also calculated.

### Region of interest (ROI) protocols

In all participants, the TFexcF, the AF, the SLF II and SLF III, the MdLF, and the ILF were reconstructed manually in the LH, with the use of DTI tractography and previously reported multiple ROI approaches, in order to provide a measure of the LH white matter damage of the language tracts in the patients examined.

For the reconstruction of the TFexcF, the protocol described in Kourtidou et al.^47^ was implemented. The SLF II, SLF III, and the AF were reconstructed according to the protocol of Barbeau and colleagues^60^. It should be noted that the AF, the axons of which link the posterior third of the STG and the immediately adjacent superior temporal sulcus with the ventrolateral frontal area 44, and to a lesser extent area 45, as well as the dorsal frontal area 8Ad^12–15^, was not always treated separately from other fasciculi, such as the SLF III, which originates from the rostral part of the inferior parietal lobule, terminating in the frontal areas 44 and 6VR. Indeed, it is not an easy case to differentiate between the axons that course around the end of the lateral fissure, forming an arch. These include monosynaptic axons of the AF, axons from the superior longitudinal fasciculus, and even axons of the middle longitudinal fasciculus (MdLF) connecting anterior and intermediate temporal regions with parietal areas (for an overview of this issue, see Petrides^11^, p. 154–165). Indeed, methodological restrictions in the study of the human white matter anatomy, have resulted in inconsistencies in the literature regarding the classification and the terminology of the dorsal white matter pathways. This has led to the emergence of different approaches in the understanding of the exact anatomy of the dorsal white matter^61^. In this context, the present study, made a choice to base the definition of the dorsal fasciculi on anatomical tracing studies on the monkey brain, later confirmed in the human brain^11–15^.Thus, in order to achieve a more accurate reconstruction, which corresponds better to the above anatomical evidence regarding these fasciculi, (i.e. AF and SLF III), the reconstruction protocols of Barbeau et al.^60^, which use exclusion ROIs in order to remove streamlines which should belong to other fasciculi (such as the MdLF or SLF II), were used in the present study.

For the MdLF, the multiple ROI approach reported by Kalyvas and colleagues^62^ was implemented, while the ILF was reconstructed according to the protocol of Wakana and colleagues^63^. Moreover, the left cingulum bundle was reconstructed for all participants as a control tract, according to the protocol of Wakana et al.^63^. Cingulum bundle is a white matter structure that connects areas from various anterior and posterior parts of the hemispheres (frontal, parietal, and medial temporal areas), as well as subcortical nuclei to the cingulate gyrus^64^. CB’s blood supply is mediated mainly by anterior and posterior cerebral artery, thus making it a good indicator of the general white matter status of the 3 middle cerebral artery CVA patients. In all tract reconstructions, streamlines terminating in brain regions which were not compatible with the known connections of each particular pathway, were excluded.. When the white matter structures were not identifiable in the color map or the FA map because of the presence of lesion, ROIs were drawn in a region homotopic on the intact hemisphere, using the available landmarks. In some cases, reconstruction of a specific tract was not possible because of the presence of a lesion in the relevant white matter, i.e. where the particular pathway courses. All DTI data were analyzed twice by a single rater (E.K.) who was unaware of the language performance of each participant, with the second analysis taking place four weeks after the initial analysis. The placement of ROIs and the reconstructed tracts was supervised by an experienced neuroanatomist (M.P.) and an experienced neuroradiologist blind to the aims of the research. The datasets of 5 participants were also analyzed by a second rater (G.A.) who was unaware of the results of the first rater. Images of the reconstructed tracts included in this study are presented in supplementary figures 2 (see Supplementary Material Section). Patients’ neuropsychological testing was also conducted within the framework of standard clinical assessment by a neuropsychologist blind to the aim of the study.

### Statistics and reproducibility

The FA values of the left TFexcF, AF, the SLF II and SLF III, the MdLF, and the ILF of the three patients were compared with the FA values of 10 control subjects, using two-sided one-sample Wilcoxon Signed Rank tests (*p* = 0.01). The FA values of the left cingulum bundle of the three patients were also compared with the FA values of the 10 controls. In each analysis, the median FA value of the healthy participants was compared with the patient’s FA index. The null hypothesis was that the control group’s median FA value will not be statistically significantly different form the critical value. In cases where the null hypothesis was rejected, we concluded that the patient’s FA is significantly deviant from the expected healthy FA. Mean values and standard deviations of the DTI metrics of the control group were calculated with the use of the IBM SPSS statistics, version 25. Results are presented in Table 1. In the present study, standard, widely used, published neuropsychological testing and publicly available software were used for behavioral and imaging data acquisition and analysis. Consequently, reproducibility is possible under specific circumstances of patients’ detection with brain lesions to specific ROIs.

### Lesion mapping approach in MNI space

The brain lesions were manually drawn in MRIcron^65^ on individual 3D T1 images in native space. The lesions were then normalized with the Clinical toolbox running in SPM12^66^, transformed into the stereotaxic Montreal Neurological Institute (MNI) space, using the MNI 152 normalization template, and were subsequently topologically defined according to the atlas of the morphology of the human cerebral cortex in the MNI Stereotaxic Space^67^. T1 weighted images were visualized using the Mango image-processing software system (http://ric.uthscsa.edu/mango/download.html). The visualization of the lesions was conducted in the MNI template, using the SurfIce (www.nitrc.org/projects/surfice).

### Reporting summary

Further information on research design is available in the Nature Research Reporting Summary linked to this article.

## Supplementary information

Supplementary Information

Reporting Summary

## Data Availability

The datasets generated and/or analyzed during the current study are not publicly available because of the inclusion of personal information that relates to identifiable individuals. Data are available from the corresponding author (E.K.) on reasonable request, with the permission of the patients included in this study.
