# Supplementary material for: Specific disruption of the ventral anterior temporo-frontal network reveals key implications for language comprehension and cognition
Source: Commun Biol. 2022 Oct 10;5:1077. doi: 10.1038/s42003-022-03983-9 (PMC9551096; doi:10.1038/s42003-022-03983-9)
Supplement: Supplementary file 1 — Supplementary Information [file 42003_2022_3983_MOESM1_ESM.pdf]

Supplementary Figure 1. Axial, coronal and sagittal MRI images of patient AA depicting the presence of lesion on BA 45, sparing the adjacent BA44.

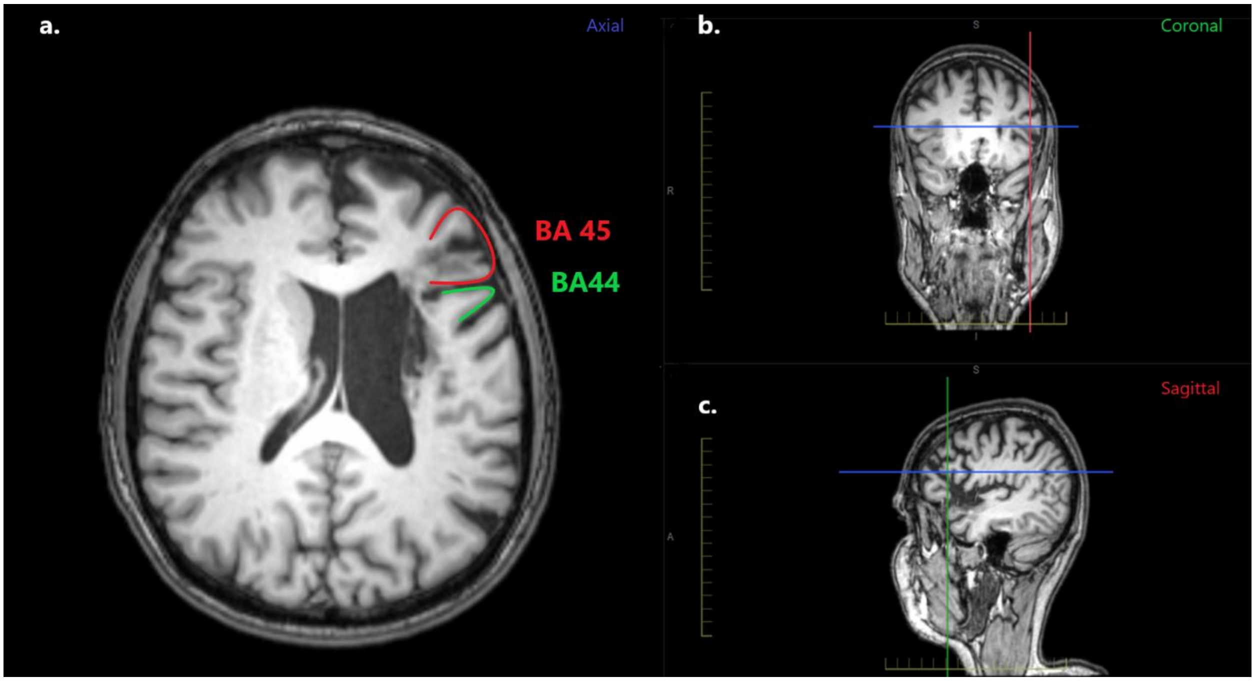

Axial (a.), coronal (b.) and sagittal (c.) view of the lesion location in Patient AA. In a. (axial view), lesioned tissue can be observed in BA 45, which is marked with red line. On the contrary, the neighboring BA 44 (marked with green line), which is usually affected along with BA 45, is intact. In b. and c., exact location of the axial, coronal and sagittal slides are represented with blue, green and red lines, correspondingly.

Supplementary Figure 2. Diffusion tensor imaging reconstruction of the white matter fasciculi included in the study for the three patients and a healthy individual.

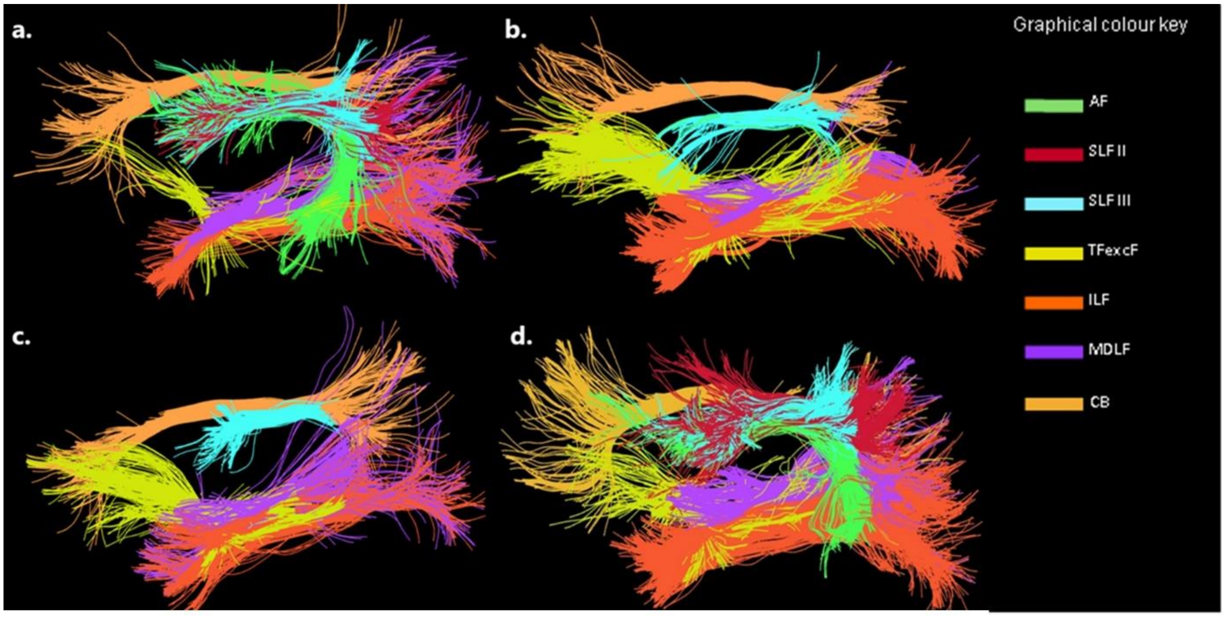

Presentation of diffusion tensor imaging (DTI) reconstructions of the arcuate fasciculus (AF; green color), superior longitudinal fasciculus II (SLF II; red color), superior longitudinal fasciculus III (SLF III; blue color), temporo-frontal extreme capsule fasciculus (TFexcF; yellow color), inferior longitudinal fasciculus (ILF; orange color), middle longitudinal fasciculus (MDLF; purple color) as well as the cingulum bundle (CB; light orange), of a., patient AA, where TFexcF was significantly reduced, b., patient MM, where SLF II reconstruction failed, SLF III was significantly affected and AF reconstruction consisted only of a few streamlines c. patient TA, where SLF II and AF reconstructions failed, while SLFIII was significantly affected, and d. a healthy participant
