# Supplementary material for: Specific disruption of the ventral anterior temporo-frontal network reveals key implications for language comprehension and cognition
Source: Commun Biol. 2022 Oct 10;5:1077. doi: 10.1038/s42003-022-03983-9 (PMC9551096; doi:10.1038/s42003-022-03983-9)
Supplement: Supplementary file 2 — Reporting Summary [file 42003_2022_3983_MOESM2_ESM.pdf]

nature portfolio

Corresponding author(s): Evie Kourtidou

Last updated by author(s): Aug 22, 2022

Reporting Summary

Nature Portfolio wishes to improve the reproducibility of the work that we publish. This form provides structure for consistency and transparency in reporting. For further information on Nature Portfolio policies, see our Editorial Policies and the Editorial Policy Checklist.

Statistics

For all statistical analyses, confirm that the following items are present in the figure legend, table legend, main text, or Methods section.

n/a	Confirmed	
☐	☒	The exact sample size (n) for each experimental group/condition, given as a discrete number and unit of measurement
☐	☒	A statement on whether measurements were taken from distinct samples or whether the same sample was measured repeatedly
☐	☒	The statistical test(s) used AND whether they are one- or two-sided<br>*Only common tests should be described solely by name; describe more complex techniques in the Methods section.*
☒	☐	A description of all covariates tested
☒	☐	A description of any assumptions or corrections, such as tests of normality and adjustment for multiple comparisons
☐	☒	A full description of the statistical parameters including central tendency (e.g. means) or other basic estimates (e.g. regression coefficient) AND variation (e.g. standard deviation) or associated estimates of uncertainty (e.g. confidence intervals)
☐	☒	For null hypothesis testing, the test statistic (e.g. F, t, r) with confidence intervals, effect sizes, degrees of freedom and P value noted<br>*Give P values as exact values whenever suitable.*
☒	☐	For Bayesian analysis, information on the choice of priors and Markov chain Monte Carlo settings
☒	☐	For hierarchical and complex designs, identification of the appropriate level for tests and full reporting of outcomes
☒	☐	Estimates of effect sizes (e.g. Cohen's d, Pearson's r), indicating how they were calculated

Our web collection on statistics for biologists contains articles on many of the points above.

Software and code

Policy information about availability of computer code

Data collection	For the MRI data collection the software that is embedded in the MRI scanner (Philips 3.0T MRI system, Achieva TX, Best, The Netherlands) was used
Data analysis	The DTI image processing and the tract reconstructions were performed with BrainanceMD (Advantis Medical Imaging, Eindhoven, The Netherlands). Brain lesions were manually drawn in MRIcron on individual 3D T1 images. Lesions were normalized and transformed into the stereotaxic MNI space with Clinical toolbox running in SPM12 and were subsequently topologically defined according to the atlas of the morphology of the human cerebral cortex in the MNI Stereotaxic Space. T1 weightened images were visualized using the Mango image-processing software system (http://ric. uthscsa.edu/mango/download.html). Brain lesions visualization was conducted in MNI space template, using the SurfIce (www.nitrc.org/projects/surfice). Mean values and standard deviations of the DTI metrics of the control group were calculated with the use of the IBM SPSS statistics, version 25.

For manuscripts utilizing custom algorithms or software that are central to the research but not yet described in published literature, software must be made available to editors and reviewers. We strongly encourage code deposition in a community repository (e.g. GitHub). See the Nature Portfolio guidelines for submitting code & software for further information.

Data

Policy information about availability of data

All manuscripts must include a data availability statement. This statement should provide the following information, where applicable:

- Accession codes, unique identifiers, or web links for publicly available datasets
- A description of any restrictions on data availability
- For clinical datasets or third party data, please ensure that the statement adheres to our policy

The datasets generated and/or analysed during the current study are not publicly available due to the inclusion of personal information that relates to identifiable individuals. Data are available from the corresponding author on reasonable request, with the permission of the patients included in this study.

Human research participants

Policy information about studies involving human research participants and Sex and Gender in Research.

Reporting on sex and gender	Three male patients were compared with ten healthy male adults
Population characteristics	Three right-handed chronic aphasia male patients, of 42, 62 and 73 years of age, with a single left hemispheric stroke and no other neurological or psychiatric diagnosis were included in this study. DTI metrics were compared with a group of right-handed healthy adults with a mean age of 54.7(9.3).
Recruitment	All healthy participants were sampled from the project "Investigation of cortical surface patterns and their relation with speech metrics and performance in neuropsychological assessment in healthy participants" conducted at Aeginition Hospital in Athens, School of Medicine, Greece (research protocol approval ID: ΩΟΞΛ46Ψ8Ν2−7ΡΝ, July 2017). Additionally, patients' data were derived from the project "Investigation of common anatomical substrate of linguistic and non-linguistic cognitive deficits in post-stroke aphasia", conducted at Aeginition Hospital in Athens, School of Medicine, Greece (research protocol approval ID: ΩΣ3Ξ46Ψ8Ν2-00Φ, July 2017).<br>To our knowledge, no bias that may in any way impact the results has taken place during the design, execution, analysis or interpretation of the data of this study.
Ethics oversight	This study was approved by the Ethics Committee of the Aeginition Hospital, Athens, Greece

Note that full information on the approval of the study protocol must also be provided in the manuscript.

Field-specific reporting

Please select the one below that is the best fit for your research. If you are not sure, read the appropriate sections before making your selection.

☒ Life sciences ☐ Behavioural & social sciences ☐ Ecological, evolutionary & environmental sciences

For a reference copy of the document with all sections, see nature.com/documents/nr-reporting-summary-flat.pdf

Life sciences study design

All studies must disclose on these points even when the disclosure is negative.

Sample size	This is a comparative three-case study. A patient with a rare lesion restricted to the ventral language network, damaging area 45 but not area 44 is examined. Additionally, two patients with lesion restricted within the classic posterior temporo-parietal region, are also examined and compared with the initial patient, providing striking dissociations and underlining the specific contribution of the ventral language pathway. The sample size (3 cases) was driven by the specific questions of the study and was based on the precise location of the lesion. Additionally, 10 right-handed healthy adults were selected from a healthy database, based on the patients' age and years of education, in order to compare the integrity of the patients' language-related fiber tracts that were intact or partially impaired by the CVA.
Data exclusions	no data were excluded from the analyses
Replication	Standard, widely used, published neuropsychological testing and publicly available softwares were used for behavioral and imaging data acquisition and analysis. Consequently, reproducibility is possible under specific circumstances of patients' detection with brain lesions to specific ROIs.
Randomization	n/a
Blinding	Lesion drawing and tract reconstruction was checked by an experienced neuroradiologist blind to the scopes of the research. Patients' neuropsychological testing was also conducted within the framework of standard clinical assessment by a neuropsychologist blind to the aim of the study.

Reporting for specific materials, systems and methods

We require information from authors about some types of materials, experimental systems and methods used in many studies. Here, indicate whether each material, system or method listed is relevant to your study. If you are not sure if a list item applies to your research, read the appropriate section before selecting a response.

Materials & experimental systems

n/a	Involved in the study	
☒	☐	Antibodies
☒	☐	Eukaryotic cell lines
☒	☐	Palaeontology and archaeology
☒	☐	Animals and other organisms
☒	☐	Clinical data
☒	☐	Dual use research of concern

Methods

n/a	Involved in the study	
☒	☐	ChIP-seq
☒	☐	Flow cytometry
☐	☒	MRI-based neuroimaging

Magnetic resonance imaging

Experimental design

Design type: The present study used structural magnetic resonance imaging (3DT1-w) and diffusion tensor imaging tractography

Design specifications: n/a

Behavioral performance measures: n/a

Acquisition

Imaging type(s): The present study used structural magnetic resonance imaging (3DT1-w) and diffusion tensor imaging tractography

Field strength: 3.0 T

Sequence & imaging parameters: The Imaging Protocol included an 1mm isotropic high resolution 3DT1-weighted sequence (time of repetition, TR)= 9.9 ms, echo time (TE)= 3.7 ms, flip angle= 70, voxel-size= 1×1×1 mm, matrix size= 244×240), an axial single shot, spin echo, echo planal DTI sequence (30 diffusion encoding directions, TR: 7299 ms, TE: 68 ms, flip angle: 900, acquisition voxel size: 2×2×2 mm, sensitivity encoding reduction factor of2, two b factors with 0 s/mm2 (low b), and 1,000 s/mm2 (high b)).

Area of acquisition: whole brain scan

Diffusion MRI: ☒ Used ☐ Not used

Parameters: 30 diffusion encoding directions, two b factors with 0 s/mm2 (low b), and 1,000 s/mm2 (high b)

Preprocessing

Preprocessing software: Brain lesions were manually drawn in mricron. 3D T1W and lesion maps in niftii format were segmented and normalized using Clinical Toolbox in SPM12 - using SPM's unified normalization-segmentation routines. All DTI pre- and post-processing was performed using BrainanceMD's (Advantis Medical Imaging, Eindhoven, The Netherlands) denoising and correction units.

Normalization: 3D T1W and lesion maps in niftii format were segmented and normalized using Clinical Toolbox in SPM12 - using SPM's unified normalization-segmentation routines. Bounding box of volume in mm: -90 -126 -72; 90 90 108. Specifically, lesion smoothing with 3mm FWHM and 0.5 threshold, binary lesion mask creation using 8mm FWMHM and 0.001 threshold.

Normalization template: MNI152

Noise and artifact removal: n/a for 3D T1-W preprocessing.

Volume censoring: n/a

Statistical modeling & inference

Model type and settings: n/a

Effect(s) tested: n/a

Specify type of analysis: ☐ Whole brain ☐ ROI-based ☒ Both

Anatomical location(s): Brain lesions were manually drawn in MRIcron5 on individual 3D T1 images in native space. Lesions were then normalized with Clinical toolbox running in SPM12, transformed into the stereotaxic MNI space and and were subsequently topologically defined according to the atlas of the morphology of the human cerebral cortex in the MNI Stereotaxic Space. DTI tractography was performed manually, based on specific protocols, reported in text, that use detailed anatomical landmarks

Statistic type for inference (See Eklund et al. 2016): voxel-wise

Correction: n/a

Models & analysis

n/a	Involved in the study
☒	☐ Functional and/or effective connectivity
☒	☐ Graph analysis
☒	☐ Multivariate modeling or predictive analysis
